# Identification and characterization of codon usage pattern and influencing factors in HFRS-causing hantaviruses

**DOI:** 10.3389/fimmu.2023.1131647

**Published:** 2023-07-10

**Authors:** Fatima Noor, Usman Ali Ashfaq, Abu Bakar, Muhammad Qasim, Muhammad Shareef Masoud, Abdulrahman Alshammari, Metab Alharbi, Muhammad Shahid Riaz

**Affiliations:** ^1^ Department of Bioinformatics and Biotechnology, Government College University, Faisalabad, Pakistan; ^2^ Centre of Agricultural Biochemistry and Biotechnology (CABB), University of Agriculture, Faisalabad, Pakistan; ^3^ Department of Pharmacology and Toxicology, College of Pharmacy, King Saud University, Riyadh, Saudi Arabia; ^4^ School of Dentistry, University of Maryland, Baltimore, MD, United States

**Keywords:** hemorrhagic fever with renal syndrome, hantavirus, RSCU, ENC, mutation, selection

## Abstract

Hemorrhagic fever with renal syndrome (HFRS) is an acute viral zoonosis carried and transmitted by infected rodents through urine, droppings, or saliva. The etiology of HFRS is complex due to the involvement of viral factors and host immune and genetic factors which hinder the development of potential therapeutic solutions for HFRS. Hantaan virus (HTNV), Dobrava-Belgrade virus (DOBV), Seoul virus (SEOV), and Puumala virus (PUUV) are predominantly found in hantaviral species that cause HFRS in patients. Despite ongoing prevention and control efforts, HFRS remains a serious economic burden worldwide. Furthermore, recent studies reported that the hantavirus nucleocapsid protein is a multi-functional protein and plays a major role in the replication cycle of the hantavirus. However, the precise mechanism of the nucleoproteins in viral pathogenesis is not completely understood. In the framework of the current study, various *in silico* approaches were employed to identify the factors influencing the codon usage pattern of hantaviral nucleoproteins. Based on the relative synonymous codon usage (RSCU) values, a comparative analysis was performed between HFRS-causing hantavirus and their hosts, suggesting that HTNV, DOBV, SEOV, and PUUV, were inclined to evolve their codon usage patterns that were comparable to those of their hosts. The results indicated that most of the overrepresented codons had AU-endings, which revealed that mutational pressure is the major force shaping codon usage patterns. However, the influence of natural selection and geographical factors cannot be ignored on viral codon usage bias. Further analysis also demonstrated that HFRS causing hantaviruses adapted host-specific codon usage patterns to sustain successful replication and transmission chains within hosts. To our knowledge, no study to date reported the factors influencing the codon usage pattern within hantaviral nucleoproteins. Thus, the proposed computational scheme can help in understanding the underlying mechanism of codon usage patterns in HFRS-causing hantaviruses which lend a helping hand in designing effective anti-HFRS treatments in future. This study, although comprehensive, relies on in silico methods and thus necessitates experimental validation for more solid outcomes. Beyond the identified factors influencing viral behavior, there could be other yet undiscovered influences. These potential factors should be targets for further research to improve HFRS therapeutic strategies.

## 1 Introduction

Hemorrhagic fever with renal syndrome (HFRS) has been a major notable epidemic mainly in Europe and Asia (1, 2). Annual cases of HFRS range from 60000 to 150000 and are highly correlated with rodent populations (3). The clinical picture is substantially characterized by a triad of symptoms including hypotension, high fever, kidney damage, and bleeding (4, 5). In different regions of the world, especially in Asia and Europe, HFRS is considered an acute zoonotic viral disease (2, 6, 7). The susceptible population of HFRS is heterogeneous in terms of geographical differences among several regions, space, as well as socioeconomic status (8). Since no therapeutic solutions are available to fight against the disease, therefore, supportive as well as symptomatic care that primarily focuses on regulating electrolyte balance, the pressure of blood and fluid might prevent complications in affected individuals (9, 10).

Several members of the hantavirus genus including Hantaan virus (HTNV) (11), Dobrava-Belgrade virus (DOBV) (12), Seoul virus (SEOV) (13), and Puumala virus (PUUV) (14) are the leading cause of HFRS. Hantaviruses are single-stranded, membrane-enveloped RNA viruses belonging to the family Hantaviridae within the order Bunyavirales (15–17). More importantly, research on therapeutic options requires a thorough understanding of their genome structure and the essential roles of their hantaviral proteins. The hantavirus genome is composed of three segments coding for G1 and G2 glycoproteins, nucleocapsid protein, and viral polymerase. Compared to non-pathogenic strains, pathogenic hantaviruses significantly alter the transcriptional activity of many cellular genes (18, 19). Recent studies provide solid evidence that hantaviruses’ nucleocapsid proteins have a key role in virus transcription, replication, and assembly (20, 21). The nucleoprotein, encoded by the S segment, of hantaviruses consist of 429 to 433 amino acids (22). This nucleoprotein interacts with the host proteins and limits the activation of the major antiviral signaling pathways in affected cells (23).

Degeneracy or redundancy of codons offers an opportunity for evolution to increase translation productivity while retaining the same sequence of amino acids (24). The codons have undergone extensive evolution, therefore, these synonymous codons are varied among species or even organisms (25). In each genome, the frequency of synonymous codons is varied and this universal event is dubbed as codon usage bias (26, 27). Molecular evolutionary studies revealed that this pattern is pervasive among eukaryotes, prokaryotes, and viruses as well as different genes of the same organism (28). Furthermore, transcriptional factors, GC content, translation, the expression level of genes, and secondary structural motifs are the main factors driving codon usage patterns (29, 30). Transcriptional factors can influence codon preferences by binding selectively to specific DNA sequences (31). The binding of these factors promotes/suppress the usage of certain codons, impacting the translation efficiency and protein production. On the other hand, genomes with higher GC content often exhibit biased codon preferences, with certain codons enriched or depleted. This bias arises from mutational processes, as well as the selective pressure for efficient translation and protein folding (32). Despite that, the main causes of the heterogeneity in codon usage among organisms are assumed to be natural selection and mutational pressure (33, 34). A slew of studies discovered that mutational pressure, rather than selection is the primary factor determining the codon usage bias (35). Additionally, mutational pressure cannot be considered as the main driving force in the case of different RNA or DNA viruses (36). Viral genomes differ from the genomes of prokaryotes and eukaryotes in certain aspects. For example, they depend on their hosts to replicate, synthesize, and transfer protein. Therefore, it is suggested that this interaction between the virus and the host affects the viral evolution, immunological escape from the host’s immune system, and viral survival (37). Host-specific selection pressures and immune-driven selection shape codon preferences in viral genes. Co-evolution between the virus and its host further impacts genetic diversity and viral adaptation (38, 39). Thus, comprehending these patterns of codon usage within viruses can help us to gain a better understanding of the genetic diversity and evolutionary dynamics in viral proteins.

This study is attributed to the identification and characterization of codon usage patterns in nucleoproteins for understanding genetic diversity and evolutionary dynamics in HTNV, DOBV, SEOV, and PUUV. Analysis of codon usage patterns would contribute to our understanding of the evolutionary mechanisms and genetic architecture of HFRS-causing hantaviruses. Thus, the present study focuses on the comprehensive analysis of genetic diversity, synonymous codon usage patterns, and factors involved in shaping the codon usage pattern of hantaviral nucleoproteins. We identified overrepresented and underrepresented codons, which could potentially be used in genetic engineering to develop better therapeutics. By leveraging the unique codon usage patterns, we can potentially optimize gene expression, enhance protein production, and improve the efficacy of antiviral treatments. Furthermore, the comprehensive analysis of genetic diversity and synonymous codon usage patterns provides valuable insights into the evolutionary mechanisms and genetic architecture of HFRS-causing hantaviruses, paving the way for future research and the development of targeted interventions.

## 2 Materials and methods

### 2.1 Data description

Obtaining coding sequences of hantaviral nucleoproteins is a preliminary step for analyzing the codon usage bias which serves as an alternative option for unveiling the evolution of HFRS-causing hantaviruses. The coding sequence of nucleocapsid proteins of HFRS-causing hantaviruses including SEOV, HTNV, DOBV, and PUUV, were retrieved from UniProt (40) with the following UniProt identifiers: P27313 (PUUV), W0LUE3 (SEOV), Q805Q9 (DOBV), and P05133 (HTNV) respectively.

### 2.2 Analysis of base composition

The coding sequences of HTNV, DOBV, SEOV, and PUUV nucleoproteins were then evaluated for the identification of nucleotide components. Two independent programs named codonW (http://codonw.sourceforge.net/) (41) and CAIcal (http://genomes.urv.es/CAIcal/) (42) were employed for calculating four different types of nucleotide frequencies at the third position including A3, U3, G3, C3, frequency of G and C at first(GC1), second(GC2) and third (GC3) positions. CodonW uses several statistical tests, such as chi-squared and Fisher’s exact tests, to determine if the observed codon usage frequencies deviate significantly from the expected frequencies (43, 44). Moreover, the UGA, UAG, and UAA are stop codon and does not code for amino acids. Therefore, UGA, UAG, and UAA codons are not anticipated to exhibit any codon usage bias and were thereby discarded from subsequent analysis.

### 2.3 Relative synonymous codon usage analysis

The Relative synonymous codon usage (RSCU) represent the codon frequency in the genome which is equal to the average frequency of all codons encoded same amino acid. CAIcal (42) software was employed for predicting RSCU within nucleoproteins of HFRS-causing viruses which in turn lends a helping hand in evaluating the codon usage bias of coding sequences. CAIcal software quantifies the similarity between the synonymous codon usage of genes and the synonymous codon frequency of a reference set and at the end measure the synonymous codon usage bias for sequence of interest (42). Codons having RSCU values above 1 represents strong bias for the corresponding codons and were referred to as ‘frequent’ codons. On the other hand, codons having RSCU value below than one show weak codon usage bias and were referred to as ‘less-frequent’ codons (45). Similarly, underrepresented and overrepresented codons were also calculated based on the RSCU value of > 1.6 and < 0.6 respectively (46). Lastly, codons having RSCU value are equal to one, representing the no codon usage bias. RSCU analysis assists in deciphering that how selection pressure and mutation shape the genetic evolution of the virus. Thus, insights into codon bias can provide valuable knowledge on viral adaptation, survival strategies, and potentially help in the design of antiviral strategies.

### 2.4 Factor influencing codon usage pattern

ENC (Effective Number codons) values were then plotted against GC3s value to analyze the effect of the overall composition of nucleotides on the synonymous codon usage pattern. The position of data points relative to the standard line in the ENC-GC3s plot provides insights into the influence of selection and mutation on codon usage. In the ENC-GC3s plot, if the observed data points are on the upper portion of the standard line, then it will indicate that the selection influences the pattern. While if the points are on the bottom part of the standard line, then it will indicate that mutation affects the pattern. So to uncover the interrelationship among ENC and GC3s values, the ENC values for codons at third positions (GC3s) were computed using the method introduced by Singh et al. (47) as follows:


$$\text{ENC}=2+s+\left(\frac{29}{s^{2}+{\left(1-s\right)}^{2}}\right)$$

Where s indicates the composition of GC3s. Further, the GC12 values (average of codons at GC1 and GC2) were then plotted against the GC3 values (48) to explore the effect of both variation and selection on the codon usage pattern of hantaviral nucleoproteins. GC12-GC3 plot examines the correlation between GC content at the first and third codon positions. A positive correlation suggests a similar pattern of variation in GC content at both positions, indicating the role of mutational bias. On the other hand, a negative correlation suggests a contrasting pattern, which can be attributed to selection.

### 2.5 Codon adaptation index analysis

The codon adaptation index (CAI) accurately predicts the potential expression levels of nucleoproteins relative to the codon usage pattern of expressed reference proteins (49). CAI measures the translation efficiency and nowadays it is used to manipulate the nucleotide sequences for maximal production of proteins to increase the process of vaccine designing. Regarding this, the CAIcal server (42) was employed to assess the CAI of HTNV, DOBV, SEOV, and PUUV nucleoproteins concerning the *Homo sapiens* host. The CAIcal server calculates the CAI value for a given gene based on the frequency of occurrence of synonymous codons for each amino acid in the gene sequence, and compares these frequencies to the reference set of highly expressed genes (50). The CAI value varies from 0 and 1 which reflects the higher levels of viral adaptation with their respective host environment.

### 2.6 Relative codon deoptimization index analysis

Recently, Mueller et al. (51) introduced the Relative codon deoptimization index (RCDI) for comparing the codon similarities between hantaviral nucleoproteins as well as host organisms. RCDI analysis provides an ultimate estimate translation rate with their respective host organism. If the pattern in both nucleoproteins and host is found to be similar, then the RCDI value is near to one which indicates excellent adaptability with their hosts. The RCDI value of nucleoproteins was computed using CAIcal (42) for finding similarities among the codon usage pattern of HTNV, DOBV, SEOV, and PUUV with *Homo sapiens*.

### 2.7 Correspondence analysis

Correspondence analysis is a multivariate statistical technique for the identification of several different variations in synonymous codon usage among proteins of interest and provides a way to figure out highly expressed genes (39). Correspondence analysis was conducted on the RSCU values to minimize the effect of inconsistencies in the overall composition of amino acids. Correspondence analysis displays a set of columns and rows in a particular dataset. The RSCU values of 59 amino acids encoding nucleoproteins were computed by representing these amino acids into 59-dimensional vectors. Each dimension in 59-dimensional vectors corresponds to the RSCU values of particular amino acids. The desired result of the correspondence analysis plot indicates the discrepancies in the usage of preferred codons among coding sequences. Additionally, by producing visual outputs, it reduces unnecessary noise and complex data structures.

### 2.8 Correlation analysis

Spearman’s rank correlation is a non-parametric measure that does not assume any particular distribution of the data and is robust to outliers. It is commonly used in codon usage analysis because it can reveal relationships between codon usage patterns and other factors that may not be linear. Spearman’s rank correlation method was employed in the current study to analyze the correlation among base contents of HTNV, DOBV, SEOV, and PUUV. If the value of the correlation coefficient (r) is 1 or near 1, then the selection is the main factor affecting the pattern of codon usage in hantaviral nucleoproteins. If the correlation among base contents is significant, the mutational pressure affects the pattern. Following that, R corrplot package was employed to conduct the correlation analysis while the codonW (41) software was employed to obtain the codon usage bias and other related indicators. R corrplot package calculate correlation coefficients and perform statistical tests to assess the significance of correlations (52). Lastly, the spearman’s rank correlation matrix was generated in R using the corrplot library which assists in the identification of the relationship between the nucleotide composition and the codon usage pattern.

### 2.9 Minimum free energy analysis

Minimum free energy was used as a measure of secondary structure formation. During the translation process, energy is released which is calculated in kcal/mol (53). A negative sign in the free energy values represents high absolute values which in turn implies a huge amount of energy loss by mRNA while adopting a stable conformation. Regarding this, RNAfold webserver (http://rna.tbi.univie.ac.at/cgi-bin/RNAWebSuite/RNAfold.cgi) (54) was used to calculate minimum free energies in hantaviral nucleoproteins. RNAfold webserver uses dynamic programming algorithm to calculate the minimum free energy of RNA secondary structures. The algorithm is based on the principles of thermodynamics and statistical mechanics, and takes into account the various energetic contributions of different base-pairing interactions in the RNA molecule (55). Later, the correlation between minimum free energy and ENC along with GC contents was then calculated to analyze the relationship among variables. In our study, we examined the correlation between minimum free energy and other variables such as ENC and GC contents. This analysis helps us explore the relationship between the stability of the secondary structure and codon usage patterns, as well as the influence of GC content on the folding potential of hantaviral nucleoproteins.

## 3 Results

### 3.1 Nucleotide compositional analysis

**Figure 1:**
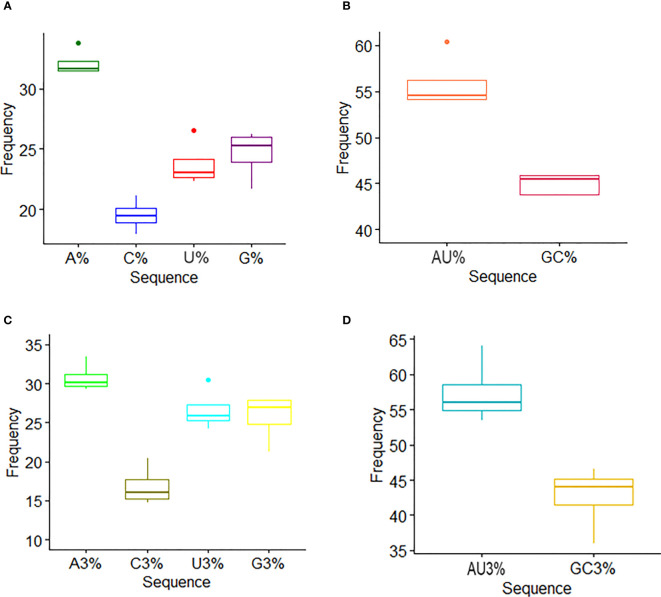
Nucleotide composition analysis. **(A)** The mean frequency for A, U, G, and C composition in nucleoprotein sequences is shown. **(B)** The mean frequency for AU and GC composition indicates AU richness. **(C)** The mean values of the nucleotide content frequency at the third codon position. **(D)** Analysis for AU and GC composition at the third codon position suggests higher AU content than GC at the third codon position.

The coding sequence of HTNV, DOBV, SEOV, and PUUV nucleoproteins was retrieved from NCBI. The composition of nucleotides in HTNV, DOBV, SEOV, and PUUV was predicted for unveiling the possible effect of nucleotide constraints on the pattern of codon usage (**Figure 1**). The percentage of A, C, T, and G was 32.2% ± 0.96, 19.5% ± 1.16, 23.7% ± 1.67, and 24.6% ± 1.7 (average ± Standard Deviation (SD)) respectively, in HFRS-causing hantaviruses. These results indicate that nucleotide A of HTNV, DOBV, SEOV, and PUUV is utilized more frequently. To get a deeper insight into the nucleotide composition in HTNV, DOBV, SEOV, and PUUV coding sequences, the mean values of the codon at the third position were further analyzed. The percentage of A3, C3, T3, and G3 was 30.7% ± 1.63, 16.8% ± 2.21, 26.6% ± 2.34, and 25.7% ± 2.74 respectively. The mean and SD of AU% and GC% contents were then calculated to be 55.8% ± 2.61 and 44.11% ± 2.60 respectively, which indicates that coding sequences of HTNV, DOBV, SEOV, and PUUV are enriched with AU% as compared to GC% nucleotides. Similarly, the mean and SD of AU3% and GC3% contents were 57.39% ± 3.98 and 42.60% ± 3.97 respectively. The composition of GC content is another important indicator to calculate the nucleotide composition bias. The average and SD of GC content at the first composition were, 51.6% ± 2.31, at the second position the average and SD were, 38.12 ± 1.80, respectively. These findings indicate that AU-ended codons are preferred at the third position. Thus, compositional constraints have a key role in the overall composition of nucleotides in the coding sequence of HTNV, DOBV, SEOV, and PUUV.

### 3.2 Analysis of relative synonymous codon usage

The RSCU value of HTNV, DOBV, SEOV, and PUUV was calculated which were then compared with the RSCU value of the host (**Table 1**). RSCU analysis revealed that 18 amino acids named UUU (Phe), UUA (Leu), AAA (Lys), GGU (Gly), CGU (Arg), UGU (Cys), GAA (Glu), GAU (Asp), AAU (Asn), CAA (Glu), UAU (Tyr), CAU (His), UCU (Ser), CCU (Pro), ACU (Thr), GCU (Ala), GUU (Val), and AUU (Ile) ended on U and A and these18 codon occupied most region of HTNV, DOBV, SEOV, and PUUV. Recent studies on equine influenza virus, avian rotaviruses, and Crimean–Congo hemorrhagic fever virus (56–58) uncovered that viruses genomes are enriched with A and U-ended codons. Additionally, the overrepresented and underrepresented codons were analyzed based on the RSCU value. Interestingly, the nucleoproteins sequences of HTNV, DOBV, SEOV, and PUUV coding sequences contained rare over-represented sequences. Only A/U-ended codons were found to be overrepresented (RSCU value > 0.6) while G/C-ended codons were found to be under-represented which unveiled that mutational pressure influences the pattern in HTNV, DOBV, SEOV, and PUUV. In the case of HTNV and SEOV, threonine has the highest RSCU value, proline has the highest RSCU value in PUUV, and alanine has the highest RSCU value in DOBV. Furthermore, the most preferred (>1.6) as well as avoided (<0.6) codons were also highlighted. Codons with an RSCU value above 1.6 were considered preferred, while those with an RSCU value below 0.6 were considered avoided. Preferred codons are those that have a higher RSCU value, indicating that they are used more frequently than other synonymous codons for the same amino acid. Conversely, avoided codons have a lower RSCU value, indicating that they are used less frequently than other synonymous codons for the same amino acid. CCC, CCG, ACG, GCG, and GCU codons were found to be avoided in HTNV, DOBV, SEOV, and PUUV. Most of the avoided codons were ended on G/C nucleotides the preferred codons were identified as A/U ended. These findings revealed that compositional pressure of nucleotides is not considered a key criterion for characterization of codon usage bias, as the RSCU value also highlighted the codon usage pattern which in turn might uncover codon usage variation in nucleoproteins of HTNV, DOBV, SEOV, and PUUV.

**Table 1:** RSCU value of amino acids and corresponding synonymous codons in HTNV, DOBV, SEOV, and PUUV.

Amino acids	Codon	HTNV	DOBV	SEOV	PUUV
Phenylalanine	UUU	1.45	1.08	0.92	1.04
	UUC	0.55	0.92	1.08	0.96
Leucine	UUA	1.4	0.67	0.86	1.43
	UUG	0.98	0.93	0.69	0.78
	CUG	1.12	1.07	1.54	0.78
	CUA	0.56	0.93	0.86	0.78
	CUU	1.12	1.33	1.37	1.43
	CUC	0.84	1.07	0.69	0.78
Isoleucine	AUU	1.4	1.39	0.93	1.59
	AUC	1	0.86	1.14	0.24
	AUA	0.6	0.75	0.93	1.16
Valine	GUU	1.22	1	0.87	1.53
	GUC	0.87	0.6	1.04	0.35
	GUA	0.35	0	0.7	1.18
	GUG	1.57	2.4	1.39	0.94
Serine	UCU	0.64	2.07	0.75	1.03
	UCC	1.07	0.21	0.19	1.24
	UCA	1.93	1.86	2.81	2.07
	UCG	0.21	0	0.38	0.21
	AGU	0.86	1.03	1.12	0.83
	AGC	1.29	0.83	0.75	0.62
Proline	CCU	1.68	1.4	2	1.08
	CCC	0	0.4	0.2	0.15
	CCA	2.11	1.8	1.4	2.62
	CCG	0.21	0.4	0.4	0.15
Threonine	ACU	0.67	0.95	1.05	1.3
	ACC	0.33	1.33	0	0.32
	ACA	3	1.52	2.95	2.16
	ACG	0	0.19	0	0.22
Alanine	GCU	0.71	0.73	1.22	0.8
	GCC	0.35	0.48	0.67	0.93
	GCA	2.94	2.55	2.11	2.27
	GCG	0	0.24	0	0
Tyrosine	UAU	1.2	0.5	1.4	1.62
	UAC	0.8	1.5	0.6	0.38
Histidine	CAU	1.67	1.14	1.71	1.25
	CAC	0.33	0.86	0.29	0.75
Glutamine	CAA	0.78	1.09	1.04	1.15
	CAG	1.22	0.91	0.96	0.85
Asparagine	AAU	0.92	1	0.92	1.57
	AAC	1.08	1	1.08	0.43
Lysine	AAA	0.83	0.94	0.48	0.94
	AAG	1.17	1.06	1.52	1.06
Aspartic acid	GAU	1.43	0.97	1.13	1.32
	GAC	0.57	1.03	0.87	0.68
Glutamic acids	GAA	0.8	1	0.96	1.07
	GAG	1.2	1	1.04	0.93
Cysteine	UGU	1.6	0.4	1.2	1.43
	UGC	0.4	1.6	0.8	0.57
Arginine	AGA	1.07	1.68	2.14	2.06
	CGA	0.64	0.24	0.21	1.03
	CGC	0.21	0.24	0.86	0.69
	CGU	0.43	0.48	0.43	0.17
	AGG	2.36	2.16	1.93	1.37
Glycine	GGU	1.23	0.48	0.74	0.71
	GGC	0.62	0.96	0.3	1
	GGA	0.77	1.6	0.89	1
	GGG	1.38	0.96	2.07	1.29

The value in yellow color represents high RSCU value (>1.6) and red color indicates low RSCU value (<0.6).

### 3.3 Factor influencing codon usage pattern

**Figure 2:**
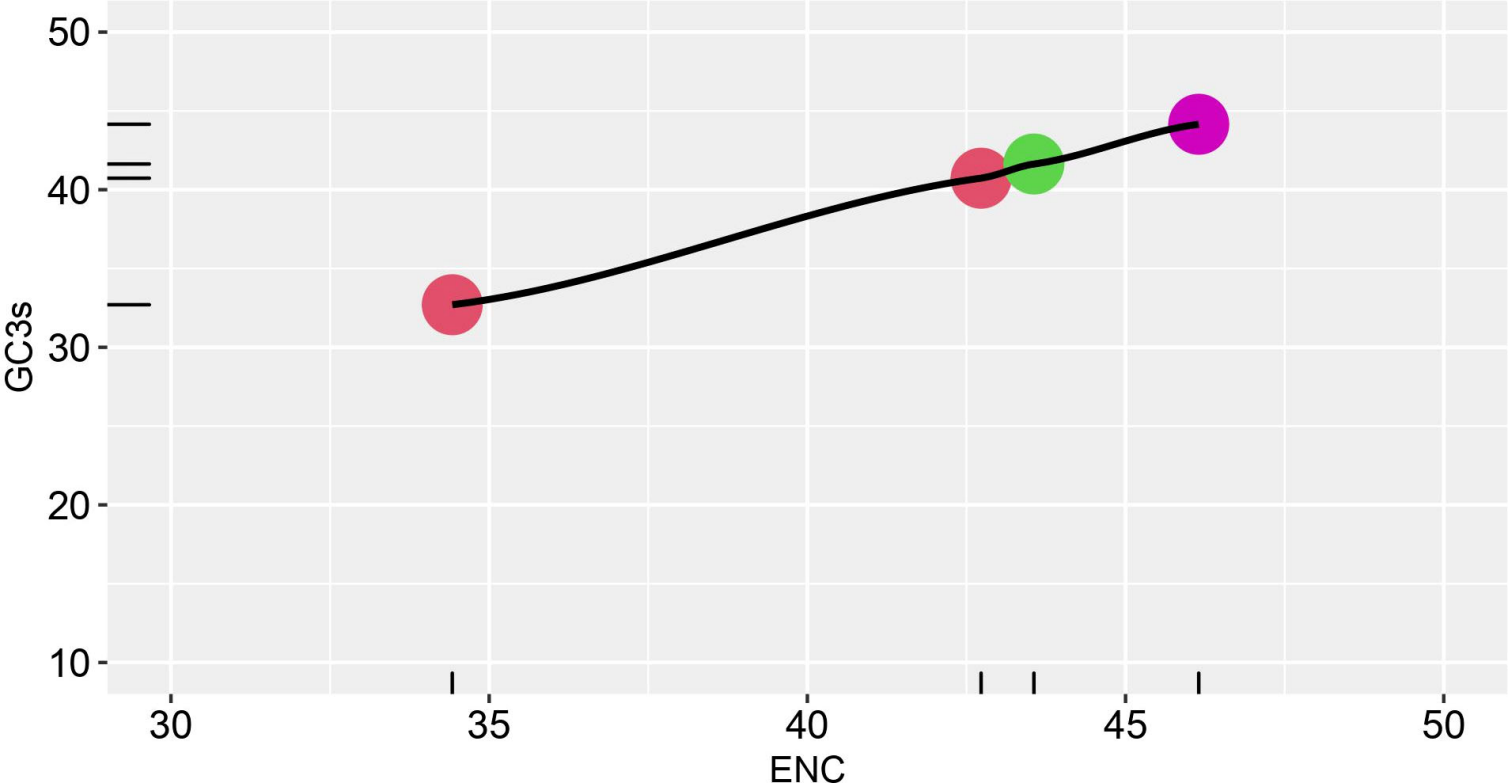
ENC-GC3 plot. The effective number of codons (ENC) plotted against the GC3S, the GC content of synonymous codons at the third position Different strains are shown in various color schemes.

To further evaluate the magnitude of codon usage patterns in HTNV, DOBV, SEOV, and PUUV, the ENC value GC3 plot was constructed. The ENC value ranges from 20 to 60. A lower ENC value represents a strong preference for codon usage while a higher ENC indicates a weaker preference for codon usage. ENC-GC3 plot helps in determining whether the pattern of codon usage in hantaviral nucleoproteins differs from that of the comparable synonymous codons. Further, the ENC value varied from 34.42 to 42.73 which represents several different trends in the codon usage pattern of HTNV, DOBV, SEOV, and PUUV nucleoproteins. **Figure 2** represents that all dots lie just below the ENC value of 60, which demonstrated that despite of selection, the variations also affect the pattern in HTNV, DOBV, SEOV, and PUUV.

**Figure 3:**
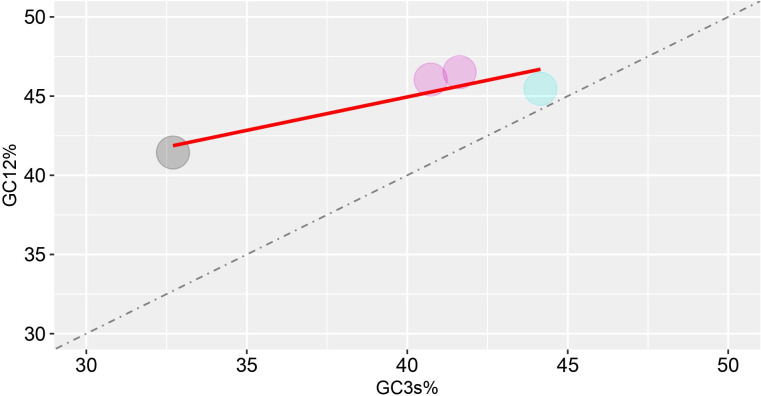
Neutrality plot analysis (GC12 vs. GC3) for the hantaviruses nucleoprotein. GC12 indicates the average value of GC contents at the first and second codon positions (GC1 and GC2), while GC3 refers to the GC contents at the third codon position.

To further analyze the natural selection as well as the mutational pressure, the GC12 were plotted against GC3 to construct the neutrality plot (**Figure 3**). In neutrality plot, each dots represent a particular virus which corresponds to the composition of GC3 and GC13 in the neutrality plot. Furthermore, if dots fall along the diagonal distribution, it reflects zero or very little pressure of external selection, and there is no discernible difference at any of the three codon positions. The variation correlation between GC12 and GC3 is instead very low if the regression curve has a tendency to be inclined or parallel to the horizontal axis. These findings of the current study revealed the correlation between GC3 and GC12 is not statistically significant (p-value = 0.1008) which does not meet the criteria of p-value > 0.05, indicating that natural selection along with mutation pressure influences the codon usage pattern of HTNV, DOBV, SEOV, and PUUV.

### 3.4 Comparative host adaptability of HFRS-causing hantaviruses

The host-pathogen relationship is the extreme case of animal association and is particularly affected by different interacting factors. The viruses co-evolved with their host and are pure parasites. A particular portion of the virus genome is associated with host-specific variations which may change the codon usage pattern of the host. Similar codon use bias is frequently seen in highly expressed viral proteins and target host proteins. So, it could be fascinating to observe how certain human parasites, like HFRS-causing hantaviruses, adapt to their hosts by reaping the benefits of the host codon usage pattern. Regarding this, the CAI values of HTNV, DOBV, SEOV, and PUUV were calculated by using *Homo sapiens* as a reference genome. The CAI value ranges from 0 to 1, with higher values indicating a better match between the codon usage of the gene and the host organism. A CAI value of 1 indicates perfect adaptation to the host organism’s codon usage, while a value of 0 indicates no adaptation. Here, the CAI value demonstrated a diverse adaptability pattern among HTNV, DOBV, SEOV, and PUUV. PUUV has a low CAI score (0.719) which is closer to SEOV (0.737), followed by HTNV (0.749), and DOBV (0.75). This phenomenon suggests that, compared to other HFRS-causing hantaviruses, DOBV and HTNV have a higher level of host adaptability.

### 3.5 Relative codon deoptimization index analysis

The coding sequences of nucleoproteins were then compared with the reference genome to figure out the similarity between the codon usage pattern of the virus with their respective hosts. Regarding this, the RCDI values were computed for comparing the codon usage pattern of HTNV, DOBV, SEOV, and PUUV with *Homo sapiens*. A higher value of RCDI represents less adaptation to the host while low RCDI values reflect higher host adaptability. RCDI value of 1 and greater than 1 represents a high translation rate which ultimately highlights a better adaptation to the host. The findings of the current study revealed the HTNV (1.367), DOBV (1.235), SEOV (1.334), and PUUV (1.379) had a host-adapted codon usage pattern as the RCDI values are equal to 1.

### 3.6 Correspondence analysis

**Figure 4:**
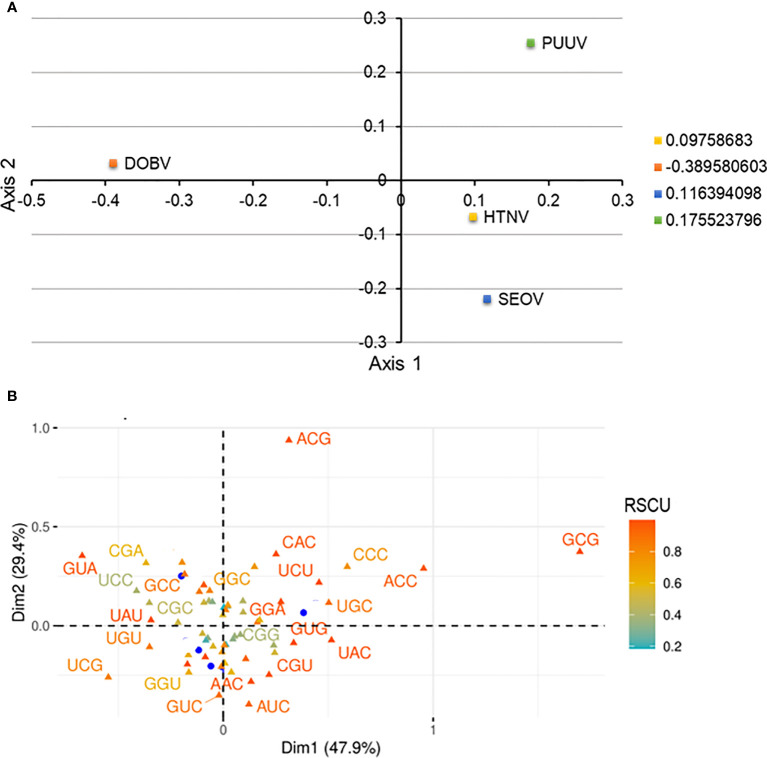
**(A)** Correspondence analysis of relative synonymous codon usage (RSCU) values of HTNV, DOBV, SEOV, and PUUV. The yellow square indicates HTNV, the blue square indicated SEOV, while the green and orange square indicates PUUV and DOBV, respectively. **(B)** The distribution of synonymous codons is shown along the first and second axes of the correspondence analysis.

Correspondence analysis is an effective and popular multivariate statistical analysis and it involves the plotting of codons in a 59-dimensional hyperspace in accordance with how frequently it uses each of 59 codons (removing start, Tryptophan, and termination codons). Correspondence analysis was conducted on RSCU values of HTNV, DOBV, SEOV, and PUUV to explore, interpret, and visualize the data. Correspondence analysis minimizes the dataset dimensions for huge multidimensional variables to visualize the different variables efficiently and effectively. HTNV, DOBV, SEOV, and PUUV were found to be dispersed using correspondence analysis (**Figure 4**). The distance among dots represents both similarities as well as dissimilarities in the pattern. The dimensions in correspondence analysis reveal the different sources of variations between a set of the multivariate data point. It is worth noting that Dimension 1 accounts for 47.9% and Dimension 2 accounts for 29.4% variations across each strain. HTNV and SEOV were found to be more closer to each other as compared to DOBV and PUUV. Altogether, these strains do not make a cluster with each other highlighting the distinction between HTNV, DOBV, SEOV, and PUUV.

### 3.7 Main constraints of the codon usage pattern

**Figure 5:**
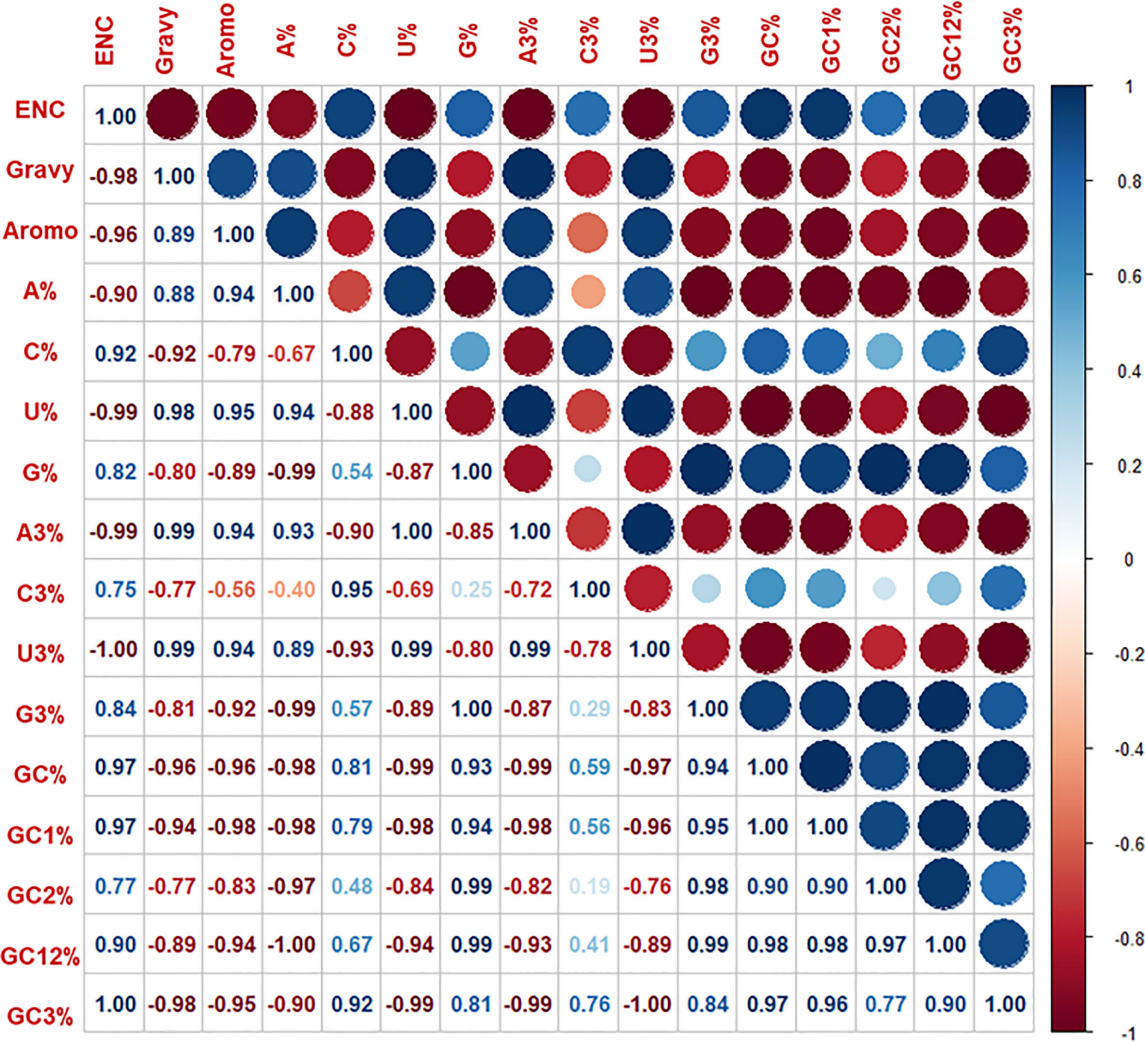
Spearman’s rank correlation matrix indicated the correlation between overall base contents and base content at the third position, ENC. Gravy and aromo. The dark blue indicates a negative correlation, and the dark red indicates a positive correlation; the higher value indicates a more significant correlation.

Mutational pressure along with selection are the two evolutionary factors which are primarily responsible for variations in the codon usage patterns. Correlation analysis of nucleotide compositions can help in the identification of primary forces causing the bias in the pattern. The overall nucleotides content (A%, U%, C%, and G%) was then correlated with the nucleotide content at the third position (A3%, U3%, C3%, and G3%) by Spearman’s rank correlation (**Figure 5**). A significant correlation was predicted at p < 0.01 and p < 0.05, representing mutational pressure as a strong force in distinguishing the codon usage bias in the nucleoproteins of HFRS-causing hantaviruses. These findings validate that apart from natural selection and mutational pressure, nucleotide composition also plays a major role in determining the shape of pattern hantaviral nucleoproteins. Further, significant correlation (correlation coefficient= 0.8, p < 0.01) was predicted among ENC and CAI values of HTNV, DOBV, SEOV, and PUUV indicating that the codon usage pattern of HFRS-causing hantaviruses is limited by both mutational pressure and natural selection.

### 3.8 Role of minimum free energy

The minimum free energy of HTNV, DOBV, SEOV, and PUUV was - 369.20, -341.50, -369.50, and -423.70 kcal/mol, respectively. The negative sign in minimum free energy represents the loss of energy during the transcription and ultimately leads to more stable conformation. A less stable structure can be formed as a result of a higher energy release, which in turn influence the translation process. Later, the minimum free energy of nucleoproteins was then correlated using Karl Pearson’s method with the ENC value of HTNV, DOBV, SEOV, and PUUV and found a significant correlation (p-value = 0.008278, r = 0.99) indicating that loss of minimum free energy was strongly related to codon usage bias. Further, the correlation between the minimum free energy of nucleoproteins and the ENC value of HTNV, DOBV, SEOV, and PUUV was calculated by Karl Pearson’s method (**Table 2**). Correlation analysis revealed that a significant correlation exists between minimum free energy and GC%, GC1%, and GC3% of HTNV, DOBV, SEOV, and PUUV nucleoproteins. On the other hand, non-significant correlations were found among minimum free energy and GC2% and GC12% of HTNV, DOBV, SEOV, and PUUV nucleoproteins. Thus, these findings indicate that minimum free energy can affect the GC compositions and that the conformation/stability of mRNA transcripts could be related to GC constraints. It is also worth noting that, one way that minimum free energy can influence codon usage bias is through its effect on mRNA stability. Stable mRNA structures can hinder the accessibility of ribosomes to the start codon and slow down translation initiation. In contrast, less stable mRNA structures are more accessible to ribosomes and promote faster translation initiation. Therefore, genes that need to be translated quickly may have a bias towards codons that promote less stable RNA structures to enhance translation efficiency. In summary, minimum free energy can affect codon usage bias by influencing mRNA stability and the speed of translation elongation. These effects can ultimately impact protein folding and function.

**Table 2:** Correlation between minimum free energy and GC contents.

Correlation	GC%	GC1%	GC2%	GC3%	GC12%
**Correlation-coffiecient**	0.93	0.93	0.68	0.99	0.84
**p-value**	0.06	0.06	0.31	0.008	0.15
**t-test**	3.74	3.59	1.33	10.84	2.23

## 4 Discussion

HFRS is a group of clinically similar illnesses caused by hantaviruses belonging to family Bunyaviridae (59). The onset of the disease is characterized by various symptoms including high fever, hemorrhage, and degree of renal insufficiency, which further evolve into coagulation disorders, acute renal failure, and anemia (4, 5). A large population of mammals and rodents harboring hantaviruses lived in towns, cities, and suburbs, therefore, now efforts are being made to avoid direct contact of hantavirus-bearing organisms with humans. Currently it is estimated that 150,000 to 200,000 cases of hantavirus disease occur each year, the majority being reported in Asia (60). Despite the high morbidity and case-fatality rates of HFRS and HCPS, respectively, no vaccine or drug is currently proven to be preventive or therapeutic. Supportive therapy is the mainstay of care for patients with hantavirus infections. Care includes careful management of the patient’s fluid (hydration) and electrolyte (e.g., sodium, potassium, chloride) levels, maintenance of correct oxygen and blood pressure levels, and appropriate treatment of any secondary infections (61). There is currently no effective treatment available for either HFRS. The most severe form of HFRS is caused by four different types of hantaviruses including HTNV, DOBV, SEOV, and PUUV (62). The nucleoproteins are more prevalent proteins in cells with viral infection and are highly conserved throughout the genus of hantavirus (63, 64). Thus, it is important to analyze the main forces causing variation in the function of hantaviral proteins which ultimately lend a helping hand in the development of novel treatment options for HFRS. More importantly, as the hantaviruses rely on the host cell’s machinery for its replication, codon usage bias could play a role in host adaptation and the virulence of the virus.

Codon usage bias is a distinctive characteristic of many organisms and has been noted in viruses such as influenza (65, 66).Relatively, there are more reports on codon usage in genomes from viruses with great harm to mankind, such as SARS, human immunodeficiency virus, influenza virus A, and hepatitis virus (67). For instance, Khattak et al. (68) conducted a genome-wide analysis of codon usage patterns for deciphering the global heterogeneity of COVID-19. Their study proposed that the general root ancestry of the global genomes is different with different genome’s level adaptations to the host. Feng et al. (69) performed a comprehensive analysis of codon usage patterns in Chinese Porcine Circoiruses (PCV) based on their major protein-coding sequences. Their findings unveiled a weak usage bias among the four PCV species and showed that in addition to mutation pressure, natural selection played a major role in PCV codon usage. Additionally, recent studies also reported that the overall pattern of codon usage in both host organisms and viruses improves the efficiency and accuracy of translation in corresponding amino acids (45, 70). While variations in the patterns ascertain the proper folding of viral nucleoproteins (71–74). Thus, analyzing codon usage patterns among HFRS-causing hantaviruses could enhance our understanding of their genetic architecture and evolutionary mechanisms. This, in turn, provide a novel insight in understanding the functioning of nucleoproteins for development of potential therapeutic strategies to fight against HFRS.

In the framework of current study, multiple systematic analytical strategies were employed to determine the factor that influences the codon usage patterns of nucleoproteins. Initially, an ENC-GC3 plot analysis was performed for analyzing the main forces behind the variation of codon usage patterns among hantaviral nucleoproteins. For this, first ENC values were calculated which were then plotted against the GC3 values. The findings proposed that ENC values are much higher as compared to ENC values of viruses causing Ebola= 57.23 (73), Crimean–Congo hemorrhagic fever= 52.34 (75), hepatitis C= 52.62) (74), and Bluetongue= 57.9) (76) indicating high bias in hantaviral nucleoproteins. A high codon usage bias affects the replication process in the host cell which increased the competition of protein synthesis in both the host and virus. Eventhough the ENC value determines the codon usage bias, these values alone does not reflect the factor that influences the codon usage pattern.

Furthermore, AT-ended codons were found to be more abundant in hantaviral nucleoproteins. Zhang et al. (77), analyzed the base content of torque tenosus virus 1 and reported A% > G% > C% > T%, suggesting the preferred use of A- over T-ending codons. Bouquet et al. (78), analyzed genetic and codon characteristics of the hepatitis E virus and reported the distribution of G and T bases to be ~ 25% each, while A was highly preferred over C. Another study conducted by Sheng et al. (67) reported the GC composition of the porcine circovirus genome to be 48.61%, playing a preferential role in synonymous codon usage. Later, to predict how these nucleotide contents altered the pattern of codon usage in nucleoproteins, we formulated our assumption from ENC–GC3 plot analysis. It indicates that mutational pressure is the main driving force contributing to the variations in the hantaviral nucleoproteins. Previous work also reported that mutational pressure influences the pattern of codon usage in Zaire ebolavirus (73). These variations are frequently constrained by the base composition at the third positions, while natural selection is mainly confined to the composition of the nucleotide at the first as well as second positions (74, 79). Thus, we conducted correlation analysis among nucleotide contents of nucleoproteins and formulated that the overall composition of nucleotide also plays important role in shaping the codon usage patterns.

To sum up, we tried to capture the characteristics of codon usage patterns in hantaviral nucleoproteins. Consequently, our study uncovered that HFRS-causing hantaviruses had a comparatively low bias and that all over-represented codons ended with A/U. Both mutational pressures, as well as natural selection, were contributors to the bias. This is the first study in investigating the codon biology in hantaviruses. Such information does not only bring about a new perspective for understanding the mechanisms of biased usage of synonymous codons but also provide useful clues for evolutionary studies. In future, our study will pave the way for designing groundbreaking approaches such as codon pair deoptimization to modify the expression efficiency of hantaviral nucleoproteins, and the usage of least preferred codons in nucleoproteins to reduce viral pathogenesis and virulence during the development of effective and safe vaccine candidate.

Codon pair deoptimization and the use of least preferred codons are strategies used to modulate the viral protein expression and attenuate viral replication. These approaches have shown promise in vaccine development by altering the codon usage patterns of viral genes, thereby reducing viral fitness and virulence. In the case of HFRS-causing hantaviruses, applying codon pair deoptimization or least preferred codon strategies could be explored as potential vaccine design strategies. By intentionally introducing suboptimal codons or reducing the usage of preferred codons in viral antigen genes, it may be possible to attenuate the replication and pathogenicity of hantaviruses, leading to the development of safer and more effective vaccines. Deoptimized viruses can express an antigenic repertoire of both B- and T-cell epitopes as they have the complete sequence of amino acids and successfully multiply *in vitro* while being highly attenuated *in vivo*, which is crucial for the development of an effective and putative vaccine. Thus, this work offers an entirely new outlook regarding genetic diversity which may contribute to the development of novel approaches for future research on the evolutionary model, their origin, and host adaptation of HFRS-causing hantaviruses.

## 5 Conclusion

Our findings proposed that analyzing variation in the codon usage pattern may offer an up-to-date strategy for unveiling the genetic diversity among hantaviral nucleoproteins. It is worth noting that mutational pressure is the main force that contributed to the variation in codon usage patterns on hantaviral nucleoproteins. The overall composition of nucleotides can also act as an influencing factor in shaping the codon usage patterns of hantaviral nucleoproteins. The current study not only provides knowledge about the variations in hantaviral nucleoproteins but also lends a helping hand in identifying the factors that drive hantavirus evolution. These findings could provide a novel way of further understanding the evolutionary changes related to viral survival, host adaptation, and virulence. The significant adaptation of hantaviral nucleoproteins to humans makes these proteins a popular indicator of evolution for HTNV, DOBV, SEOV, and PUUV host speciation, and may assist in understanding the epidemic character of HFRS in humans. The information from this research may not only be helpful to get new insights into the evolution of hantaviruses but also have potential value for developing novel vaccines to fight against HFRS. Additionally, it might improve our understanding of how nucleoproteins function in HFRS and shed light on the therapeutic options. Such findings will be conducive to understanding the elements that contribute to viral evolution and adaptation to hosts.

## Data availability statement

The original contributions presented in the study are included in the article/supplementary material. Further inquiries can be directed to the corresponding author.

## Author contributions

FN, UAA, and MQ contributed to the conception and design of the study. AB, MSM, MA, and MSR collected the data. FN performed the analysis. FN and UAA wrote the first draft of the manuscript. AB, MQ, MSM, AA, MA, and MSR revised the manuscript. All authors contributed to the article and approved the submitted version.
